# A systematic review and standardized clinical validity assessment of male infertility genes

**DOI:** 10.1093/humrep/dez022

**Published:** 2019-03-13

**Authors:** Manon S Oud, Ludmila Volozonoka, Roos M Smits, Lisenka E L M Vissers, Liliana Ramos, Joris A Veltman

**Affiliations:** 1 Department of Human Genetics, Donders Institute for Brain, Cognition and Behavior, Radboud University Medical Centre, Nijmegen, The Netherlands; 2 Institute of Genetic Medicine, Newcastle University, Newcastle, United Kingdom; 3 Scientific Laboratory of Molecular Genetics, Riga Stradins University, Riga, Latvia; 4 Department of Obstetrics and Gynecology, Division of Reproductive Medicine, Radboud University Medical Centre, Nijmegen, The Netherlands

**Keywords:** male infertility, spermatogenic failure, genetics, clinical validity, gene–disease relation, gene panel, next-generation sequencing, systematic review

## Abstract

**STUDY QUESTION:**

Which genes are confidently linked to human monogenic male infertility?

**SUMMARY ANSWER:**

Our systematic literature search and clinical validity assessment reveals that a total of 78 genes are currently confidently linked to 92 human male infertility phenotypes.

**WHAT IS KNOWN ALREADY:**

The discovery of novel male infertility genes is rapidly accelerating with the availability of next-generating sequencing methods, but the quality of evidence for gene–disease relationships varies greatly. In order to improve genetic research, diagnostics and counseling, there is a need for an evidence-based overview of the currently known genes.

**STUDY DESIGN, SIZE, DURATION:**

We performed a systematic literature search and evidence assessment for all publications in Pubmed until December 2018 covering genetic causes of male infertility and/or defective male genitourinary development.

**PARTICIPANTS/MATERIALS, SETTING, METHODS:**

Two independent reviewers conducted the literature search and included papers on the monogenic causes of human male infertility and excluded papers on genetic association or risk factors, karyotype anomalies and/or copy number variations affecting multiple genes. Next, the quality and the extent of all evidence supporting selected genes was weighed by a standardized scoring method and used to determine the clinical validity of each gene–disease relationship as expressed by the following six categories: no evidence, limited, moderate, strong, definitive or unable to classify.

**MAIN RESULTS AND THE ROLE OF CHANCE:**

From a total of 23 526 records, we included 1337 publications about monogenic causes of male infertility leading to a list of 521 gene–disease relationships. The clinical validity of these gene–disease relationships varied widely and ranged from definitive (*n* = 38) to strong (*n* = 22), moderate (*n* = 32), limited (*n* = 93) or no evidence (*n* = 160). A total of 176 gene–disease relationships could not be classified because our scoring method was not suitable.

**LARGE SCALE DATA:**

Not applicable.

**LIMITATIONS, REASONS FOR CAUTION:**

Our literature search was limited to Pubmed.

**WIDER IMPLICATIONS OF THE FINDINGS:**

The comprehensive overview will aid researchers and clinicians in the field to establish gene lists for diagnostic screening using validated gene–disease criteria and help to identify gaps in our knowledge of male infertility. For future studies, the authors discuss the relevant and important international guidelines regarding research related to gene discovery and provide specific recommendations for the field of male infertility.

**STUDY FUNDING/COMPETING INTEREST(S):**

This work was supported by a VICI grant from The Netherlands Organization for Scientific Research (918-15-667 to J.A.V.), the Royal Society, and Wolfson Foundation (WM160091 to J.A.V.) as well as an investigator award in science from the Wellcome Trust (209451 to J.A.V.).

**PROSPERO REGISTRATION NUMBER:**

None.

## Introduction

Infertility is defined as the inability to conceive within 1 year of unprotected sexual intercourse (Zegers-Hochschild *et al.*, 2009). Approximately 7% of the male population is affected, collectively explaining infertility in half of all couples affected (Irvine, 1998; Winters and Walsh, 2014; Krausz and Riera-Escamilla, 2018).

The etiology of infertility is highly heterogeneous, which is not surprising when considering that both male and female reproductive systems need to function in a combined and precisely coordinated fashion in order to conceive a child. Studies aiming to elucidate the genetic basis of fertility defects in both human and mice have defined numerous crucial pathways for male infertility, including sexual differentiation, development of the genitourinary system and gametogenesis (Jamsai and O’Bryan, 2011; Krausz and Riera-Escamilla, 2018). Currently more than 600 male infertility genes have been described in the Jackson Laboratory’s Mouse Genome Informatics database (http://www.informatics.jax.org/) and 2300 testis-enriched genes are currently known in human (Schultz *et al.*, 2003). High-impact mutations in any of these genes will always remain at very low frequency in the population because of their impact on fitness. This means that in order to find recurrently mutated genes and novel genes confidently linked to infertility one has to screen large cohorts of patients for pathogenic variants in large numbers of genes. This has been laborious and expensive for a long time due to limitations of traditional genetic assays such as Sanger sequencing. Since the first introduction of next-generation sequencing (NGS) in 2005, the technology has evolved to allow rapid and affordable sequencing of large amounts of DNA (Metzker, 2010). This has expedited sequencing of large gene panels, all coding genes (the exome) and even whole genomes (Payne *et al.*, 2018).

### Genetic testing in infertility

It is currently thought that at least 15% of all human male infertility patients can be explained by genetic defects (Krausz and Riera-Escamilla, 2018). Since the discovery of an extra X chromosome in Klinefelter patients (47,XXY) (Ferguson-Smith *et al.*, 1957; Jacobs and Strong, 1959), more than 3500 papers have been published on the genetics of male infertility, implicating various common genetic origins as well as hundreds of other genes in male infertility. Despite these large numbers, genetic diagnostic testing is usually confined to karyotyping, azoospermia factor (AZF) deletion screening and cystic fibrosis transmembrane conductance regulator (*CFTR)* mutation analysis.

Currently a genetic diagnosis is reached in about 4% of all infertile males – a number that has not increased since the late 1990s (Johnson, 1998; Tuttelmann *et al.*, 2018). This is in sharp contrast to the increase in diagnostic yield seen for other conditions with a strong genetic component, driven largely by the widespread application of genomic microarray analysis and NGS (Rehm, 2017; Tuttelmann *et al.*, 2018). Without a genetic diagnosis it is difficult for a clinician to counsel couples with questions about the causes of their infertility, possible co-morbidities, the potential success of ART and the reproductive health of their offspring.

### Clinical validity assessment of gene–disease relationships

With the introduction of and advances in genomics, the number of genes associated with male infertility has expanded in recent years. However, the amount of genes confidently linked to disease is still very limited in comparison to developments in other genetic diseases such as intellectual disability (Vissers *et al.*, 2016; Tuttelmann *et al.*, 2018). This is caused in part by a lack of solid evidence linking variation in individual genes to human male infertility. The notion of sub-optimal quality of evidence in male infertility research is not limited to genetic studies but is considered a general concern in the field of reproductive biology (Evers, 2013; Barratt, 2016; Glujovsky *et al.*, 2016).

In order to robustly link gene dysfunction to disease, one needs to consider multiple levels of evidence. This is especially important since insufficient, inconclusive and low-quality evidence may result in incorrect and misleading conclusions about gene–disease relationships. Moreover, if this wrongful gene–disease relation is not identified and corrected, it may lead to inappropriate diagnoses and even mismanagement and counseling of the infertile couples involved. Furthermore, these incorrectly characterized genes may complicate follow-up research by contaminating candidate disease gene lists and pathway analyses.

Recently, the Clinical Genome Resource (ClinGen) has developed an extensive framework to assess the clinical validity of a gene–disease relationship (Strande *et al.*, 2017). However, the overall number of validated disease genes is currently very limited (*n* = 490) and does not contain any genes involved in male infertility. Another more simplified and pragmatic version of this framework was recently published to more easily assess the clinical validity of gene–disease relationships (Smith *et al.*, 2017). In the present study, we applied the gene–disease scoring system of Smith *et al.* (2017) to curate all available information on the genetics of human male infertility from 1958 up to December 2018. This analysis allowed us to objectively classify the evidence for the involvement of genes in male infertility as non-existing, limited, moderate, strong or definitive. The results from this work may be useful in both research and diagnostics, for example for developing diagnostic gene panels and hopefully help to strengthen genetic research in male infertility.

## Materials and Methods

### Search strategy and study selection

Two independent reviewers conducted a literature search in Pubmed according to the PRISMA guidelines for English articles in peer-reviewed journals. The search was performed on several occasions with the last search taking place on 6 December 2018 without further restrictions on publication date. The search query and screening strategy aimed to collect all records of genetics research in defective male reproductive development and function (Supplementary Table SI). Doubts about inclusion of any publications were resolved by discussion and consensus between all authors.

### Data extraction and assessment of clinical validity

From eligible papers presenting original data, we extracted the gene names, patient phenotypes, inheritance pattern, method of discovery and whether or not single nucleotide or copy number variants (CNV) were identified in the genes mentioned in infertile men. After extraction of the gene names from all records, we employed a recently published gene–disease scoring system to establish the strength of evidence for the relationship between a gene and male infertility (Smith *et al.*, 2017). A detailed description of the evidence assessment and an assessment template are described in Supplementary Tables SII and SIII. Similar to the publication selection process, disagreements and debatable cases were resolved by consensus between all authors. In order to prevent bias in gene–disease evaluation, a second and a third reviewer independently reviewed and verified a random selection of 12 and 16 gene–disease relationships, respectively.

### Overview of biological knowledge

From all genes with at least limited evidence, we also extracted the reported or expected results of semen analysis (if available), whether the patients described are sporadic or familial cases, and whether the type of infertility was isolated, a reproductive organ syndrome, endocrine disorder or part of another syndrome. All genes with at least limited evidence were plotted according to their biological function.

## Results

### Search strategy and study selection

**Figure 1:**
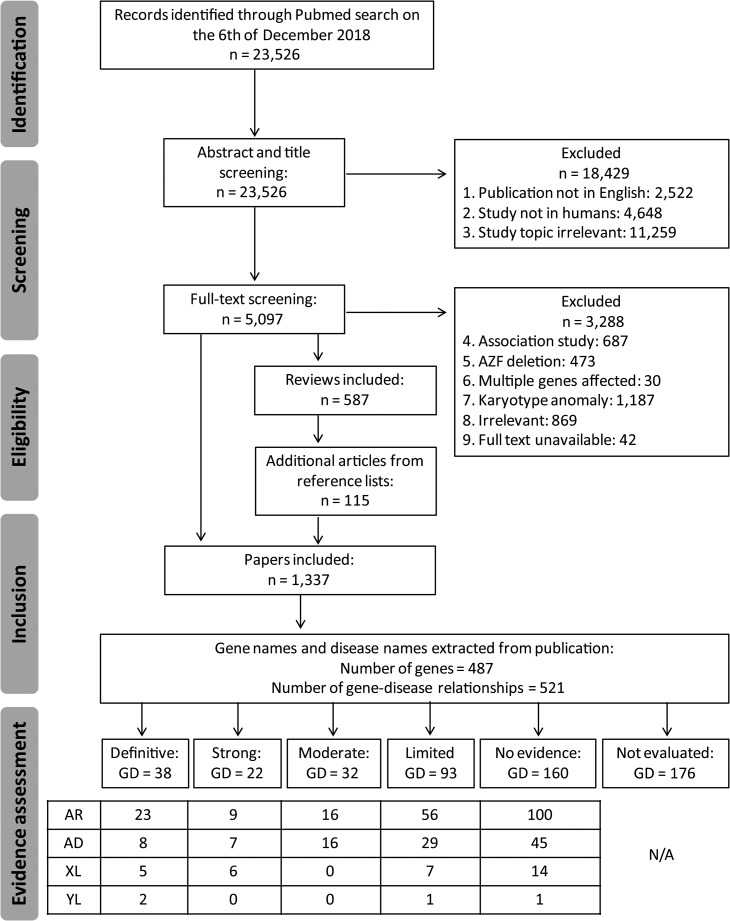
**PRISMA flow chart showing our search and screening strategy to identify publications and genes eligible for clinical validity assessment in male infertility.** GD, gene–disease relationships; AR, autosomal recessive; AD, autosomal dominant; XL, X-linked; YL, Y-linked.

With our search strategy, we aimed to identify all publications covering the genetics of male infertility, including those underlying syndromes affecting the endocrine system, disorders of sex development and genitourinary anomalies. Our search yielded a grand total of 23 526 publications that date from 1958 to 2018 (Fig. 1). Based on title and abstract, 18 429 studies were excluded because the publication was not in written English or the study topic did not match our inclusion criteria (Supplementary Table SI). Although severe syndromes including male infertility phenotypes were excluded because affected patients are unlikely to seek help to reproduce because of severe physical or intellectual disabilities, we included milder syndromes and syndromes affecting the reproductive organs only. A total of 5097 publications were left. Since the scope of our systematic review is monogenic male infertility, we then excluded papers that described genetic association or risk factors (*n* = 687), AZF deletions (*n* = 473), CNVs affecting multiple genes (*n* = 30) or chromosomal anomalies (*n* = 1187). In addition, we excluded 869 publications that, based on full-text analysis, were not covering the topic of the genetics of male infertility and we excluded 42 papers for which the full text was unavailable. We then screened the reference lists from included reviews (*n* = 587) and were able to add another 115 publications that were not identified by our search strategy. In total, our search yielded 1337 publications that met our inclusion criteria (Fig. 1).

**Figure 2:**
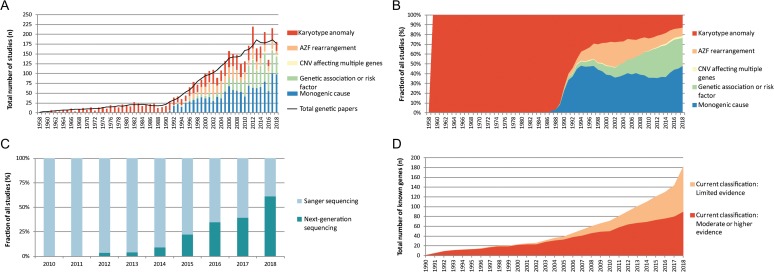
**Genetic studies in male infertility between 1958 and 2018.** (**A**) Graphical overview of genetic studies in male infertility. (**B**) Graphical representation of type of genetic research in male infertility. (**C**) The use of Sanger sequencing and next-generation sequencing for the discovery of genes in male infertility. (**D**) Increase of genes linked to human male infertility. AZF, azoospermia factor; CNV, copy number variants.

The systematic literature search revealed a total of 150–200 publications per year in the past 10 years and showed that the majority of publications from the last few years report on monogenic causes of male infertility (46% in 2017), followed by genetic association or risk factor analysis (28% in 2017) (Figs 2A and B). Furthermore, the absolute number of karyotype studies has been relatively stable over the past 20 years at ~30 publications per year.

### Data extraction and evaluation of evidence

From the 1337 included publications, we extracted 487 unique Human Genome Organization (HUGO) approved gene names and 521 gene–disease relationships (Fig. 1). The number of gene–disease relationships is higher than the number of genes because several genes were described in multiple male infertility phenotypes. A further look into the discovery method showed that DNA sequencing has been the most commonly used technique for novel gene discovery and replication studies (84% of all publications). At the moment, a shift from Sanger sequencing to NGS methods is taking place (Fig. 2C).

We then assessed the clinical validity of each gene–disease relationship by using the simplified scoring system designed to establish the strength of a relationship between a single gene and a Mendelian disease (Smith *et al.*, 2017) (Supplementary Tables SII and SIII).

After excluding genes that did not contain any potentially pathogenic variant or were unable to be classified, a total of 345 gene–disease relationships were curated and classified into the following categories: definitive (*n* = 38), strong (*n* = 22), moderate (*n* = 32), limited (*n* = 93) and no evidence (*n* = 160) (Supplementary Tables SIV and SV). We identified a total of 78 genes that can at least be moderately linked to a total of 92 male infertility or abnormal genitourinary development phenotypes showing autosomal recessive (*n* = 48), autosomal dominant (*n* = 31), X-linked (*n* = 11) and Y-linked (*n* = 2) inheritance patterns. Patients were found to be sporadic (*n* = 18), in families (*n* = 14) or in both (*n* = 60) and led to isolated infertility (*n* = 24), a reproductive organ or endocrine syndrome (*n* = 55) or a syndromic form of infertility (*n* = 13) (Table I; Supplementary Table SIV). In 176 cases, we could not evaluate the gene–disease relationship because either the inheritance pattern remains unclear or suggests polygenic inheritance, the technical quality of the identification method was too poor or the exact variant information could not be retrieved (Supplementary Table SVI).

**Table I:** Numbers of genes that are at least moderately linked to male infertility or abnormal genitourinary development phenotypes.

Description	AR	AD	XL	YL	Total
Type of infertility					
Isolated infertility	16	4	4	0	24
Syndromic infertility	9	3	1	0	13
Endocrine disorder/Reproductive system syndrome	23	24	6	2	55
Testicular phenotype					
Sertoli cell-only syndrome	2	0	0	0	2
Pre-meiotic arrest	0	1	0	0	1
Meiotic arrest	2	3	1	0	6
Spermiogenesis defect	16	1	1	0	18
Unknown stage or multiple stages affected	3	3	1	0	7

AR, autosomal recessive; AD, autosomal dominant; XL, X-linked; YL, Y-linked.

The results show that the total number of confidently linked genes is growing steadily at about three genes per year. The increase in NGS methods being used has caused an exponential growth in novel candidate genes. However, the vast majority of these are currently classified as ‘Limited evidence’. In the past 5 years, 84 new disease genes have been described of which only 27% (*n* = 23) have now been at least moderately linked to male infertility leaving 73% (*n* = 61) with a current classification of ‘Limited evidence’ (Fig. 2D).

### Overview of human genes involved in human male infertility

**Figure 3:**
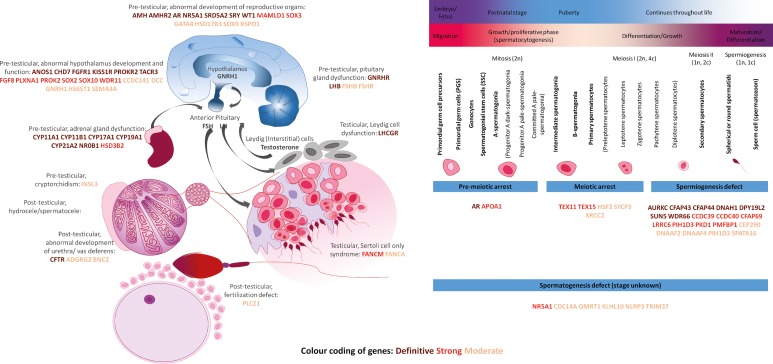
**Biological overview of the genetics of male infertility.** The color of each gene indicates the amount of evidence: Brown: Definitive; Red: Strong; Orange: Moderate. GnRHR: GnRH receptor, LHCGR: LHCG receptor. A list of gene names and definitions is available in Supplementary Table SIX.

Taking into account that normal functioning of the male reproductive system is biologically mostly dictated by the hypothalamic–pituitary–gonadal axis, the origins of male infertility can be divided in three major groups: pre-testicular, testicular and post-testicular. We grouped all genes with at least limited evidence for an involvement in human male infertility into these three groups based on their reported biological function (Fig. 3) to assess whether the curated genes play a role in these biological processes.

Our results show that pre-testicular forms of infertility are mostly syndromic and caused by endocrine abnormalities, characterized by low levels of sex steroids and abnormal gonadotrophin levels. Post-testicular causes include ejaculatory disorders or obstructions, which impair the transport of spermatozoa from the testis. These obstructions can be caused by a congenital unilateral or bilateral absence of the vas deferens.

Despite the fact that monomorphic forms of teratozoospermia are extremely rare, half of all genes known to cause isolated testicular forms of infertility are involved in teratozoospermia (*n* = 10, 50% of all 20). The number of genes confidently linked to the more common phenotypes oligozoospermia or azoospermia when mutated remains limited (*n* = 10, 50% of all 20).

## Discussion

This standardized clinical validity assessment focused on the genetic causes of infertility and provides a systematic and comprehensive overview of all genes implicated as a monogenic cause of male infertility. Our study aimed to provide an overview of all currently available evidence and gene–disease relationships, as well as formulate a set of recommendations for future studies involving the genetics of male infertility.

### Clinical validity of gene–disease relationships in male infertility

In our literature search, we used a simplified version of the extensive framework used by ClinGen to curate gene–disease relationships and results in similar evidence categories. The method was previously described and proved to be reliable, reproducible and similar to the conclusions of the ClinGen method, which makes the method suitable for robust and rapid evaluation of genes in both research and diagnostic sequencing settings (Smith *et al.*, 2017). In general, the results of our clinical validity assessment demonstrate that the quality of evidence for gene–disease relationships as well as the reporting of the results varies greatly in the field of male infertility.

The results of this clinical validity assessment are not static and as knowledge increases over time the outcome may be subjected to changes over time. Hence, we expect that a large number of the genes that are currently classified as ‘Limited’, ‘No evidence’ or ‘Unable to classify’ may still play an important role in male infertility and should therefore not be omitted from future genetic studies.

Evidence from animal models was often strong and genetic studies clearly benefit from a wealth of studies describing hundreds of well-characterized male infertility mouse models (de Boer *et al.*, 2015; Kherraf *et al.*, 2018). However, caution is urged in drawing conclusions about gene function and inheritance mode based on mouse models only. The mouse and human reproductive system are not identical and genes may have (slightly) different functions or transmit disease through different modes of inheritance (Lieschke and Currie, 2007). For this reason, we included statistical evidence from large human datasets to supplement the evidence from animal models (Lek *et al.*, 2016; Quinodoz *et al.*, 2017). In case the evidence for inheritance pattern was clearly contradictory between mice and human, we did not evaluate the gene–disease relationship (*n* = 92).

The number of candidate gene–disease relationships is growing exponentially as a result of the availability of NGS methods and 2018 has already yielded more novel gene–disease relationships than 2015, 2016 and 2017 combined (Fig. 2D). However, the number of confidently linked gene–disease relationships is not growing at the same pace. The major reason for this is that most genes have only been found mutated in single patients and functional evidence is lacking. We expect the number of genes confidently linked to azoospermia to grow in the coming years by large-scale data sharing, especially since this is a common form of infertility and genetic components are very likely to play an important role in its etiology (Krausz and Riera-Escamilla, 2018).

### Importance of re-evaluation of evidence

The recent availability of large genetic population reference databases facilitates re-evaluation of reported disease-associated variants and allows for determining whether the population frequency of the variant is in line with a reported link to a disorder associated with reduced fitness such as male infertility. Previous reports have shown that healthy participants on average have ~54 exonic variants that were previously reported to be pathogenic, but based on their allele frequency were likely to be misclassified (Lek *et al.*, 2016).

The systematic re-classification of reported genetic sequencing variants in male infertility using this information resulted in some interesting observations. For example, protein interacting with PRKCA 1 (*PICK1)* is regularly mentioned as a gene that causes globozoospermia in human patients (De Braekeleer *et al.*, 2015; Ray *et al.*, 2017; Krausz and Riera-Escamilla, 2018). However, only one patient with one homozygous variant has ever been described in an initial report of a Chinese globozoospermia patient and no new patients have been published since (Liu *et al.*, 2010). With the current gnomAD database available, we now know that 1.74% of the East Asian population carries the variant (http://gnomad.broadinstitute.org/variant/22-38471068-G-A). Globozoospermia is an extremely rare type of male infertility and the expected allele frequency of a globozoospermia-causing variant is at least 60-fold lower than the observed allele frequency of this variant. It is therefore highly unlikely that this particular variant is causing globozoospermia in this patient, based on the allele frequency.

Despite the gene–disease relation being based on the wrong data, PICK1 deficiency has been shown to result in disruption of acrosome formation in mice and *PICK1* is expressed in human testis (Xiao *et al.*, 2009). Hence, based on these observations the gene remains an important candidate gene for human male infertility. Similar discrepancies in originally published allele frequencies and currently available allele frequencies were found in several other genes including NLR family pyrin domain containing 14 (*NLRP14)* (http://gnomad.broadinstitute.org/variant/11-7060977-A-T) (Westerveld *et al.*, 2006), septin 12 (*SEPT12)* (http://gnomad.broadinstitute.org/variant/16-4833970-C-T) (Lin *et al.*, 2012) and Rhox homeobox family member 1 (*RHOXF1)* (http://gnomad.broadinstitute.org/variant/X-119243190-C-T) (Borgmann *et al.*, 2016).

Next to the availability of genetic population reference databases, recently a number of detailed human testis transcriptome studies were published (Jan *et al.*, 2017; Li *et al.*, 2017; Guo *et al.*, 2018; Hermann *et al.*, 2018). These studies are of tremendous value to better understand the role of genes in spermatogenesis and help to better classify the evidence for male infertility gene–disease relationships.

### Recommendations for genetic testing in male infertility

During our study, we noted that international guidelines for nomenclature and interpretation of sequencing variants were often not followed even long after the introduction and worldwide acceptation of these guidelines (Richards *et al.*, 2015; den Dunnen *et al.*, 2016). We identified several errors in nomenclature of sequencing variants and in some cases the variants were not named in a meaningful and unequivocal manner rendering them unusable for assessment. Furthermore, many publications did not mention the expected or proven inheritance pattern or reached doubtful conclusions about the mode of inheritance.

In order to ensure efficient sharing and downstream use of newly identified sequencing variants and genes, it is crucial to report variants in an unambiguous and standardized way. In adherence to the standard American College of Medical Genetics guidelines, we have made a list of recommendations for future reporting of novel male infertility variants (Supplementary Table SVII). Furthermore, our literature study shows that the quality of evidence of a gene–disease relationship varies greatly. We recommend the use of public and local genomic reference databases, and statistical and functional experiments to build evidence for causality (Supplementary Table SVIII).

### The genetics of human male infertility: overview and future perspectives

Our work shows that the field of genetics of male infertility is rapidly expanding due to the introduction of NGS methods (Fig. 2). However, currently, of all 521 gene–disease relationships described, only 18% (*n* = 92 gene–disease relationships involving 78 genes) have been at least moderately linked to the disease and an additional 18% (*n* = 93 gene–disease relationships involving 86 genes) are candidate gene–disease relationships with only limited evidence for involvement of the gene in a male infertility phenotype (Supplementary Table SIV; Fig. 3). Caution is warranted when using genes with limited or no evidence for diagnostic screening.

Similar to other fields in medical genetics, the field of genetics in male infertility has largely focused on inherited variation. Our analysis indicates that 52% of all gene–disease relationships with at least moderate evidence for an involvement in male infertility show an autosomal recessive inheritance pattern (*n* = 48 of 92 gene–disease relationships involving 45 genes) (Table I). Importantly, many of these genes have been identified in consanguineous families and are unlikely to play an important role in infertility in the outbred population. Secondly, many of these genes are associated with very specific and rare sperm defects. It is therefore unlikely that these genes will play a major role in the more common quantitative sperm defects (azoospermia and oligozoospermia) encountered in outbred populations. In contrast, our analysis revealed that only 34% of all gene–disease relationships (*n* = 31 of 92, involving 24 genes) with at least moderate evidence for causing male infertility has an autosomal dominant inheritance pattern (Table I), most of which are syndromic presentations.

It may perhaps not be surprising that there is only a limited number of autosomal dominant genes described for male infertility, as pathogenic variation in these genes can only be passed through the maternal line. Importantly, however, studies in intellectual disability and developmental delay have recently pointed to an important role for *de novo* germline mutations resulting in autosomal dominant disease (Vissers *et al.*, 2016). The *de novo* mutation hypothesis for male infertility is further underscored by the fact that *de novo* chromosomal and structural variations are well-known causes of male infertility: Klinefelter syndrome and AZF deletions almost exclusively occur *de novo* (Lanfranco *et al.*, 2004; Colaco and Modi, 2018). The role of *de novo* point mutations, however, remains unexplored in male infertility so far. At the moment, only three autosomal dominant genes are moderately linked to isolated male infertility: doublesex and mab-3 related transcription factor 1 (*DMRT1*), kelch like family member 10 (*KLHL10*) and synaptonemal complex protein 3 (*SYCP3*). Unfortunately, parental samples were not studied for any of these genes to find out whether the variant was paternally or maternally inherited or occurred *de novo*.

### Genetic testing in diagnostic settings

The recommendations for genetic testing during the diagnostic work-up of male infertility patients have only minimally changed over the last 20 years and most of these recommendations still focus on the well-known and common causes of male infertility that were already known in the 1990s (Barratt *et al.*, 2017; Jungwirth *et al.*, 2018). For cost-efficiency, there are guidelines to help stratify patient groups to receive pre-conceptive genetic tests such as karyotype analysis, AZF deletion tests or a screening for pathogenic variants in a single gene involved in a specific phenotype such as Kallmann syndrome. However, after stratification, in ~40% of all male infertility patients no genetic cause is found with the above mentioned tests (Krausz and Riera-Escamilla, 2018) and this strongly suggests that much more genetic research is required and at the same time the use of other diagnostic assays should be considered.

Testing all patients for all genetic anomalies was very costly for a long time. However, in light of the recent developments of novel sequencing technologies, it is now possible to consolidate one or multiple tests in a single NGS assay, which will help to cut the costs. The first examples of NGS-based screening methods have been described for male infertility (Oud *et al.*, 2017; Fakhro *et al.*, 2018; Patel *et al.*, 2018). The European Society of Human Genetics (ESHG) and ESHRE have recently made a recommendation for developing and introducing new tests, specifically for extended carrier screening (Harper *et al.*, 2018). The identification of novel disease genes allows for the selection of genes for male infertility gene panels. For diagnostic purposes, gene panels should contain genes with a minimal level of evidence of involvement with disease. We recommend including genes with an evidence classification of at least ‘Moderate’ for the composition of diagnostic gene panels. While it is difficult to predict at this moment how much the diagnostic yield of genetic testing will increase for the different subtypes of male infertility, it is realistic to expect the overall yield to go from the current 4% (Tuttelmann *et al.*, 2018) to more than 10% in the coming decade.

## Conclusion

In this clinical validity assessment, we evaluated a total of 521 gene–disease relationships involving 487 genes with reported monogenic association to male infertility and identified 92 gene–disease relationships with at least moderate evidence for a role in male infertility. Our results as well as our objective approach and recommendations may aid the robust and rapid identification and incorporation of novel genes in male infertility diagnostics.

## Supplementary Material

Supplementary Table 1

Supplementary Table 2

Supplementary Table 3-6

Supplementary Table 7

Supplementary Table 8

Supplementary Table 9
