# Supplementary material for: A systematic review and standardized clinical validity assessment of male infertility genes
Source: Hum Reprod. 2019 Mar 13;34(5):932–41. doi: 10.1093/humrep/dez022 (PMC6505449; doi:10.1093/humrep/dez022)
Supplement: Supplementary Table 1 [file dez022_supplementary_table_si.pdf]

Human Reproduction, 2019
doi:10.1093/humrep/dez022

human reproduction

SUPPLEMENTARY DATA

Supplementary Table SI **Search strategy, inclusion and exclusion criteria.**

**Search term Pubmed**	'Infertility, Male/genetics'[Mesh]<br>OR<br>'Male sterility due to Y-chromosome deletions' [Supplementary Concept]<br>OR<br>(('genetics'[MeSH Terms] OR 'genetics'[Subheading] OR 'genetics'[All Fields] OR 'genetic'[All Fields] OR 'Genetic Variation'[Mesh] OR 'mutation'[All Fields] OR 'mutations'[All Fields] OR 'polymorphism'[All Fields] OR 'polymorphisms'[All Fields] OR 'Karyotype'[Mesh] OR 'Karyotype'[All fields] OR 'Chromosomes, Human, Y/genetics'[Mesh] OR (('Y chromosome'[All Fields] OR 'chromosome Y'[All Fields] OR 'Y-chromosome'[All Fields]) AND 'microdeletion'[All Fields]) OR 'AZFc deletion'[All Fields] OR 'AZFb deletion'[All Fields] OR 'AZFa deletion'[All Fields] OR 'gr/gr deletion'[All Fields])<br>AND<br>('infertility, male'[MeSH Terms] OR ('infertility'[All Fields] AND 'male'[All Fields]) OR 'male infertility'[All Fields] OR ('male'[All Fields] AND 'infertility'[All Fields]) OR 'infertile men'[All Fields] OR 'azoospermia'[MeSH Terms] OR 'azoospermia'[All Fields] OR 'Sertoli Cell-Only Syndrome'[MeSH] OR 'Sertoli Cell-Only'[All Fields] OR 'Aspermia'[All Fields]<br>OR<br>'oligospermia'[MeSH Terms] OR 'oligospermia'[All Fields] OR 'oligozoospermia'[All Fields] OR 'cryptozoospermia'[All Fields] OR 'hypospermatogenesis'[All Fields] OR 'spermatogenic failure'[All Fields] OR 'asthenozoospermia'[MeSH Terms] OR 'asthenozoospermia'[All Fields] OR 'Asthenospermia'[All Fields] OR 'multiple morphological abnormalities of the sperm flagella '[All Fields] OR 'MMAF'[All Fields] OR 'Dysplasia of the fibrous sheath'[All Fields] OR 'Kartagener Syndrome'[Mesh] OR 'Kartagener Syndrome'[All Fields] OR 'Primary ciliary dyskinesia'[All Fields] OR 'teratozoospermia'[MeSH Terms] OR 'teratozoospermia'[All Fields] OR 'teratospermia'[All Fields] OR 'globozoospermia'[All Fields] OR 'macrozoospermia'[All Fields] OR ('acephalic'[All Fields] AND ('spermatozoa'[MeSH Terms] OR 'spermatozoa'[All Fields])) OR 'Congenital bilateral aplasia of vas deferens' [Supplementary Concept] OR 'Congenital bilateral aplasia of vas deferens'[All Fields] OR 'Congenital bilateral absence of vas deferens'[All Fields] OR 'CBAVD'[All Fields] OR 'Vas Deferens/abnormalities'[Mesh] OR 'Cryptorchidism'[Mesh] OR 'Cryptorchidism'[All Fields] OR 'Persistent Mullerian duct syndrome'[Supplementary Concept] OR 'Persistent Mullerian duct syndrome'[All Fields] OR 'Hypospadias'[Mesh] OR 'Hypospadias'[All Fields]<br>OR<br>'Kallmann Syndrome'[Mesh] OR 'Kallmann Syndrome'[All fields] OR 'Hypogonadism'[Mesh] OR 'Hypogonadism'[All fields] OR 'Klinefelter Syndrome'[Mesh] OR 'Klinefelter Syndrome'[All fields] OR 'XXY'[All fields] OR '46, XY Disorders of Sex Development'[Mesh] OR '46, XX Disorders of Sex Development'[Mesh] OR '46, XY Disorders of Sex Development'[All Fields] OR '46,XY Disorders of Sex Development'[All Fields] OR '46, XX Disorders of Sex Development'[All Fields] OR '46,XX Disorders of Sex Development'[All Fields] OR '46, XY Disorder of Sex Development'[All Fields] OR '46,XY Disorder of Sex Development'[All Fields] OR '46, XX Disorder of Sex Development'[All Fields] OR '46,XX Disorder of Sex Development'[All Fields] OR 'Androgen-Insensitivity Syndrome'[Mesh] OR 'androgen insensitivity syndrome'[All fields] OR 'Leydig Cell Hypoplasia' [Supplementary Concept] OR 'Receptors, LH'[Mesh] OR 'Leydig cell hypoplasia'[All Fields] OR (('Gonadal Dysgenesis/genetics'[Mesh] OR 'Gonadal Dysgenesis, 46,XY'[Mesh] OR 'Gonadal Dysgenesis' [All Fields]) AND ('46,XY'[All Fields] OR '46, XY'[All Fields] OR '46XY'[All Fields])))<br>NOT<br>('Plant Infertility'[Mesh] OR 'Fungi'[Mesh] OR 'Cattle'[Mesh] OR 'Swine'[Mesh] OR 'Intellectual Disability'[Mesh] OR ('Heart Defects, Congenital'[Mesh] NOT ('Dextrocardia'[Mesh] OR 'Kartagener Syndrome'[Mesh] OR 'Noonan Syndrome'[Mesh] OR 'Turner Syndrome'[Mesh] OR 'Heterotaxy syndrome'[Mesh])) OR 'Leukoencephalopathies'[Mesh] OR 'Preimplantation Diagnosis'[Mesh] OR 'Prenatal Diagnosis'[Mesh] OR 'Leukemia'[Mesh] OR 'Histocompatibility Antigens Class I'[Mesh] OR 'Histocompatibility Antigens Class II'[Mesh]))<br>AND<br>1900:2018/12/06[dp]
**Inclusion criteria**	• Publish in peer-reviewed journal<br>• Studies concentrating on male infertility and/or defective genitourinary development<br>• Studies conducted in human patients<br>• Studies concentrating on finding a genetic cause<br>• Studies presenting a monogenic form of infertility<br>• Studies presenting original data

Continued

Supplementary Table SI *Continued*

**Exclusion criteria title/ abstract screening**	• Publication not in English<br>• Studies investigating animals/plants/fungi/prokaryotes<br>• Study topic is irrelevant and/or includes one of the following examples:<br>– Female infertility<br>– Studies investigating polygenic or multifactorial forms of infertility<br>– Sperm aneuploidies or chromosomal anomalies without mentioning constitutional chromosomal anomalies<br>– Sperm DNA fragmentation or damage<br>– mtDNA copy load<br>– Variants described in population databases<br>– Epigenetics/methylome<br>– Sperm mRNA expression<br>– Studies about patients with Prader scale 0–3 (included: scale 4, 5 and 6)<br>– Syndromes characterized by severe physical or intellectual disability including CHARGE, Werner, Noonan, 4H, Opitz G/BBB, Gordon Holmes, Nijmegen breakage, Cabezas, MEHMO, Dilated Cardiomyopathy and Ataxia, Boucher-Neuhauser, Hartsfield syndrome, Aarskog-Scott, Huppke-Brendel, Brooks, Juberg-Marsidi, Warburg Micro, Prune Belly<br>– Other syndromes: Thalassemia, Myhre, Waardenburg, Bardet-Biedl, Wilms Tumor, Alström, Complete Androgen Insensitivity syndrome, Swyer syndrome, Frasier syndrome, Woodhouse-Sakati, Cystic Fibrosis
**Exclusion criteria full text screening**	• Studies investigating genetic risk factors or associations without presuming a direct consequence of the variant on the gene/protein function<br>• Deletion of multiple genes in AZF regions<br>• Deletion or duplication of multiple genes<br>• Studies presenting chromosomal aneuploidies or rearrangements without charging a (set of)affected gene(s)<br>• Full text reveals study topic is irrelevant (based on exclusion criterion 3)<br>• Full text not available
