# Supplementary material for: A systematic review and standardized clinical validity assessment of male infertility genes
Source: Hum Reprod. 2019 Mar 13;34(5):932–41. doi: 10.1093/humrep/dez022 (PMC6505449; doi:10.1093/humrep/dez022)
Supplement: Supplementary Table 2 [file dez022_supplementary_table_sii.pdf]

Supplementary Table SII SOP classification gene–disease relationships.

Required programs

Alamut Visual 2.10

Required databases

DOMINO: https://wwwfbm.unil.ch/domino/index.html
ExAC: http://exac.broadinstitute.org/
GnomAD: http://gnomad.broadinstitute.org/
OMIM: https://www.omim.org/
MGI: http://www.informatics.jax.org/
STRING: https://string-db.org/
Protein Atlas: https://www.proteinatlas.org/
In-house database 3347 proven fathers (Radboudumc)

Required protocols

Original protocol Smith *et al.*:
https://www.ncbi.nlm.nih.gov/pubmed/28106320
ACMG variant classification guidelines:
https://www.acmg.net/ACMG/Publications/Laboratory_Standards___Guidelines/ACMG/Publications/Laboratory_Standards___Guidelines.aspx?hkey=8d2a38c5-97f9-4c3e-9f41-38ee683bcc84
ClinGen Gene Curation SOP Version 5:
https://www.clinicalgenome.org/site/assets/files/8891/gene_curation_sop_2016_version_5_11_6_17.pdf

Procedure

- Inheritance pattern
  - (a) Note the inheritance pattern that was given by the authors
  - (b) Check DOMINO, ExAC and OMIM for expected inheritance pattern in human
  - (c) Check MGI and/or publications for inheritance pattern seen in model organisms (such as mouse)
  - (d) Make conclusion based on all evidence (AR/AD/XL/YL/Other/Undetermined)
  - (e) Write in notes if inheritance pattern conclusion does not match the inheritance pattern given in publication
  - (f) If inheritance pattern is not AD/AR/XL/YL (e.g. mtDNA/polygenic), classify as 'Unable to classify' and move on to next gene–disease relationship.
  - (g) Otherwise continue with Step 2–10
- Retrieve the information about the patients. Only assess male patients with a Prader scale of stage 4 or higher (looks more male than female)
  - (a) In case of syndromes like Kallmann syndrome or Primary Ciliary Dyskinesia, only include male patients that have strong evidence for infertility (so proven hypogonadotropic hypogonadism or proven asthenozoospermia).
  - (b) Do not include female patients with the same syndrome
- Retrieve all coding and/or splice site variants in male patients alone (no female patients) and open them in Alamut
  - (a) Check the allele frequency in control databases (at least GnomAD and in-house database proven fathers Radboudumc)
    - (i) Except for variants in *CFTR* which may be more common due to founder effects in North European populations (Bombieri *et al.*, 2015), we used a maximum allele frequency of 1% in the general population as a threshold value. Variants causing fully penetrant monogenic severe male infertility suffer from strong selection in the general population and are unlikely to reach higher allele frequencies than 1% (Eilbeck *et al.*, 2017). Variants that were more common were classified as (likely) benign. Next to various freely available population databases such as GnomAD (Lek *et al.*, 2016), we also used an anonymized local database with exome variants found in 3347 fathers of children who have been referred for trio Whole Exome Sequencing (WES) in the Radboud University Medical Centre. The healthy fathers reflect the general Dutch population and to our knowledge conceived naturally.
  - (b) Classify the variant according to the ACMG system. Take into account functional assays
  - (c) If variant cannot be retrieved from data or the detection quality is insufficient, classify as 'Unable to classify' and move on to next gene.
  - (d) If multiple possibly damaging variants in different genes are present in the same patient, classify as 'Unable to classify'
  - (e) Otherwise continue with Step 4–11
- For each unrelated proband (family counts as 1), write down the number of patients with a VUS/Likely pathogenic/Pathogenic variant.
  - (a) If no variants are found, all variants are (likely) benign, or the variant does not match the inheritance pattern (e.g. heterozygous variant in autosomal recessive disease) classify as 'No evidence' and move on to next gene.
  - (b) Otherwise continue with Step 5–11
- In case of family: search in text for LOD score. If this is not available use the Gene Curation SOP version 5 11_6_17.
  - (a) If higher than 3, assign 1 point for Other Statistical Evidence
    - (i) If no LOD score was given, we use a simplified formula as provided by the Clinical Genome Resource Gene Curation Working Group (Strande *et al.*, 2017).
    - (ii) For dominant/X-linked diseases: $Z\,(LOD\ score) = \log 10 \frac{1}{0.5^{\text{segregations}}}$
    - (iii) For recessive diseases: $Z\,(LOD\ score) = \log 10 \frac{1}{0.25^{\#\ \text{affected individuals}-1}\, 0.75^{\#\ \text{unaffected individuals}}}$.
- In case of de novo variants: search in text for statistical analysis for excess of de novos in disease.
  - (a) If significant, assign 1 point for Other Statistical Evidence
- For each (likely) pathogenic variant (so VUS excluded), assign 1 point for Number of Pathogenic Variants.

Continued

Supplementary Table SII *Continued*

	• Search in text for information about gene expression.<br>(a) If not available, use Protein Atlas (Uhlen *et al.*, 2015), the Testis Transcriptional Atlas (Guo *et al.*, 2018) or search in Pubmed for other publications.<br>(b) Assign 1 point for Gene Function if function/expression is consistent with phenotype<br>• Search in text for information about protein interactions.<br>(a) If not available, use STRING (Szklarczyk *et al.*, 2017) or search in Pubmed for other publications.<br>(b) Assign 1 point for Gene Function if interaction is consistent with phenotype<br>• Search in text for information about *in vitro* studies<br>(a) If not available, search in Pubmed for other studies on this gene<br>(b) Critically evaluate methods and results of *in vitro* studies. If doubtful, do not assign points<br>(c) Assign 1 point for relevant pathology *in vitro* after similar genetic modification<br>(d) Assign 1 point for determination of mutational mechanism<br>• Search in text for information about model organisms<br>(a) If not available, search in MGI (Smith *et al.*, 2018) or in Pubmed for publications<br>(b) Assign 1 point for gene function *in vivo* related to pathology of human disease<br>(c) Assign 1 point for phenotype and genotype match human disease<br>• Repeat Step 2–11 for each publication about a gene–disease relationship.<br>• After the final publication, assign points for the number of unrelated patients:<br>(a) 1 pt: 1–2, 2 pt: 3–4, 3 pt: 5–9, 4 pt: 10–24 patients<br>• After the final publication, assign points for the number of replication studies describing unrelated probands with at least a VUS. The first publication should be considered as the index patient and points should therefore only be counted from the second publication onwards.<br>• Determine the classification 0 pt: No evidence, >2 pt: Limited, >8 pt: Moderate, >12 pt: Strong, >15 pt: Definitive
**Validation by independent reviewer**	• Randomly assign a selection of gene–disease relationships to an independent reviewer. Collect the results<br>• For comparison between reviewers the following ranges in points are acceptable: 0–4 pt: No evidence, 2–9 pt: Limited, 8–12 pt: Moderate, >12 pt: Strong, >15 pt: Definitive<br>• If the difference in assigned points is larger than 1, the reviewers discuss the discrepancies.<br>• Repeat process if necessary
