# Supplementary material for: A systematic review and standardized clinical validity assessment of male infertility genes
Source: Hum Reprod. 2019 Mar 13;34(5):932–41. doi: 10.1093/humrep/dez022 (PMC6505449; doi:10.1093/humrep/dez022)
Supplement: Supplementary Table 7 [file dez022_supplementary_table_svii.pdf]

Supplementary Table SVII **Recommendations for reporting of sequencing variants.**

Topic	Recommendation
Variant calling	To ensure reliable results, establish the variant calling accuracy of the technique used and only report variants that meet a minimal level of accuracy
Variant discovery	All relevant variants should be validated in an independent sequencing reaction to prevent reporting of false-positive results
Gene nomenclature	Use the standard HGNC nomenclature:<br>https://www.genenames.org/about/guidelines
Variant nomenclature	Use the standard HGVS nomenclature:<br>http://varnomen.hgvs.org/
Provide sequence reference	All reported variants should include:<br>• Genome build (e.g. GRCh37/hg19)<br>• Transcript ID (e.g. NM_198393.3)
Inheritance pattern	Report the expected or proven inheritance pattern. Note that the inheritance pattern between mice and human may be discordant<br>Public databases containing inheritance information:<br>OMIM (Human): https://www.omim.org/<br>MGI (Mouse): http://www.informatics.jax.org/<br>ClinGen (Human): https://search.clinicalgenome.org/kb/gene-validity/<br>Prediction of inheritance pattern:<br>Domino: https://wwwfbm.unil.ch/domino/index.html
Classification of sequencing variants	If a gene has been linked to male infertility, apply the ACMG guidelines to classify the sequencing variants.<br>ACMG guidelines: https://www.acmg.net/docs/ACMG%20SG%20Interpretation%20of%20Sequence%20Variants%20GIM%20May%202015%20(3).pdf
