# Supplementary material for: A systematic review and standardized clinical validity assessment of male infertility genes
Source: Hum Reprod. 2019 Mar 13;34(5):932–41. doi: 10.1093/humrep/dez022 (PMC6505449; doi:10.1093/humrep/dez022)
Supplement: Supplementary Table 8 [file dez022_supplementary_table_sviii.pdf]

Supplementary Table SVIII **Recommendations for providing evidence for gene–disease relationships.**

Topic	Recommendation
Population frequency	Public databases can be used with care. Please note that public databases contain female samples and infertile men. Local databases and/or healthy controls are preferred if available.<br>Examples of public databases:<br>• GnomAD: http://gnomad.broadinstitute.org/<br>• Exome Variant Server: http://evs.gs.washington.edu/EVS/<br>• dbSNP: https://www.ncbi.nlm.nih.gov/SNP/
Segregation analysis	Testing of parents and other family members preferred to establish if identified variant segregates with disease or occurred *de novo*
Recurrence in affected individuals	Other patients with similar phenotypes should be tested for variants in same gene. If no patients are available, collaborative efforts and matchmaking services are recommended.<br>Example of matchmaking service:<br>Matchmaker Exchange: https://www.matchmakerexchange.org/
Statistical evidence	Gene burden analysis can be used to proof that a gene shows a statistical excess of rare variants in a patient cohort compared to control cohorts.
In silico prediction	Prediction models may be used to filter variants that are likely to be deleterious.Examples for missense variants:<br>• SIFT: http://sift.jcvi.org/<br>• MutationTaster: http://www.mutationtaster.org/<br>• PolyPhen2: http://genetics.bwh.harvard.edu/pph2/<br>Examples for splice variants:<br>• GeneSplicer: http://ccb.jhu.edu/software/genesplicer/<br>• Human Splicing Finder: http://www.umd.be/HSF3/<br>• MaxEntScan:http://genes.mit.edu/burgelab/maxent/Xmaxentscan_scoreseq.html
Gene expression	Report if the newly identified gene is expressed in relevant tissue(s). Expression can be studied in patient/healthy control material or through public databases.<br>Examples of public databases:<br>Protein Atlas: https://www.proteinatlas.org/<br>Expression Atlas: https://www.ebi.ac.uk/gxa/home
Protein interaction	Report if newly identified gene interacts with known disease genes. Interaction can be studied *in vitro* or through public databases.<br>Example of public database:<br>STRING: https://string-db.org/
Functional validation	Prove disruptive nature of identified sequencing variant in patient cells or in an *in vitro* model
In vivo evidence	The phenotype of an animal model recapitulates the human phenotype. Preferably rescue experiments are performed.
