# Supplementary material for: A systematic review and standardized clinical validity assessment of male infertility genes
Source: Hum Reprod. 2019 Mar 13;34(5):932–41. doi: 10.1093/humrep/dez022 (PMC6505449; doi:10.1093/humrep/dez022)
Supplement: Supplementary Table 9 [file dez022_supplementary_table_six.pdf]

Supplementary Table SIX Gene names and definitions of genes at least moderately linked to male infertility (Fig. 3).

Gene symbol	Gene definition	HUGO Gene Nomenclature ID
ADGRG2	adhesion G protein-coupled receptor G2	HGNC:4516
AMH	anti-Mullerian hormone	HGNC:464
AMHR2	anti-Mullerian hormone receptor type 2	HGNC:465
ANOS1	anosmin 1	HGNC:6211
APOA1	apolipoprotein A1	HGNC:600
AR	androgen receptor	HGNC:644
AURKC	aurora kinase C	HGNC:11391
BNC2	basonuclin 2	HGNC:30988
CCDC141	coiled-coil domain containing 141	HGNC:26821
CCDC39	coiled-coil domain containing 39	HGNC:25244
CCDC40	coiled-coil domain containing 40	HGNC:26090
CDC14A	cell division cycle 14A	HGNC:1718
CEP290	centrosomal protein 290	HGNC:29021
CFAP43	cilia and flagella associated protein 43	HGNC:26684
CFAP44	cilia and flagella associated protein 44	HGNC:25631
CFAP69	cilia and flagella associated protein 69	HGNC:26107
CFTR	cystic fibrosis transmembrane conductance regulator	HGNC:1884
CHD7	chromodomain helicase DNA binding protein 7	HGNC:20626
CYP11A1	cytochrome P450 family 11 subfamily A member 1	HGNC:2590
CYP11B1	cytochrome P450 family 11 subfamily B member 1	HGNC:2591
CYP17A1	cytochrome P450 family 17 subfamily A member 1	HGNC:2593
CYP19A1	cytochrome P450 family 19 subfamily A member 1	HGNC:2594
CYP21A2	cytochrome P450 family 21 subfamily A member 2	HGNC:2600
DCC	DCC netrin 1 receptor	HGNC:2701
DMRT1	doublesex and mab-3 related transcription factor 1	HGNC:2934
DNAAF2	dynein axonemal assembly factor 2	HGNC:20188
DNAAF4	dynein axonemal assembly factor 4	HGNC:21493
DNAH1	dynein axonemal heavy chain 1	HGNC:2940
DPY19L2	dpy-19 like 2	HGNC:19414
FANCA	FA complementation group A	HGNC:3582
FANCM	FA complementation group M	HGNC:23168
FGF8	fibroblast growth factor 8	HGNC:3686
FGFR1	fibroblast growth factor receptor 1	HGNC:3688
FSHB	follicle stimulating hormone subunit beta	HGNC:3964
FSHR	follicle stimulating hormone receptor	HGNC:3969
GATA4	GATA binding protein 4	HGNC:4173
GNRH1	gonadotropin releasing hormone 1	HGNC:4419
GNRHR	gonadotropin releasing hormone receptor	HGNC:4421
HS6ST1	heparan sulfate 6-O-sulfotransferase 1	HGNC:5201
HSD17B3	hydroxysteroid 17-beta dehydrogenase 3	HGNC:5212
HSD3B2	hydroxy-delta-5-steroid dehydrogenase, 3 beta- and steroid delta-isomerase 2	HGNC:5218
HSF2	heat shock transcription factor 2	HGNC:5225
INSL3	insulin like 3	HGNC:6086
KISS1R	KISS1 receptor	HGNC:4510
KLHL10	kelch like family member 10	HGNC:18829
LHB	luteinizing hormone beta polypeptide	HGNC:6584
LHCGR	luteinizing hormone/choriogonadotropin receptor	HGNC:6585

Continued

Supplementary Table SIX *Continued*

Gene symbol	Gene definition	HUGO Gene Nomenclature ID
LRRC6	leucine rich repeat containing 6	HGNC:16725
MAMLD1	mastermind like domain containing 1	HGNC:2568
NLRP3	NLR family pyrin domain containing 3	HGNC:16400
NR0B1	nuclear receptor subfamily 0 group B member 1	HGNC:7960
NR5A1	nuclear receptor subfamily 5 group A member 1	HGNC:7983
PIH1D3	PIH1 domain containing 3	HGNC:28570
PKD1	polycystin 1, transient receptor potential channel interacting	HGNC:9008
PLCZ1	phospholipase C zeta 1	HGNC:19218
PLXNA1	plexin A1	HGNC:9099
PMFBP1	polyamine modulated factor 1 binding protein 1	HGNC:17728
PROK2	prokineticin 2	HGNC:18455
PROKR2	prokineticin receptor 2	HGNC:15836
RSPO1	R-spondin 1	HGNC:21679
SEMA3A	semaphorin 3A	HGNC:10723
SOX10	SRY-box 10	HGNC:11190
SOX2	SRY-box 2	HGNC:11195
SOX3	SRY-box 3	HGNC:11199
SOX9	SRY-box 9	HGNC:11204
SPATA16	spermatogenesis associated 16	HGNC:29935
SRD5A2	steroid 5 alpha-reductase 2	HGNC:11285
SRY	sex determining region Y	HGNC:11311
SUN5	Sad1 and UNC84 domain containing 5	HGNC:16252
SYCP3	synaptonemal complex protein 3	HGNC:18130
TACR3	tachykinin receptor 3	HGNC:11528
TEX11	testis expressed 11	HGNC:11733
TEX15	testis expressed 15, meiosis and synapsis associated	HGNC:11738
TRIM37	tripartite motif containing 37	HGNC:7523
WDR11	WD repeat domain 11	HGNC:13831
WDR66	WD repeat domain 66	HGNC:28506
WT1	Wilms tumor 1	HGNC:12796
XRCC2	X-ray repair cross complementing 2	HGNC:12829
